# Weight loss is associated with improved daytime time in range in adults with prediabetes and non-insulin-treated type 2 diabetes undergoing dietary intervention

**DOI:** 10.1111/dme.70052

**Published:** 2025-06-03

**Authors:** Souptik Barua, Dhairya Upadhyay, Lauren T. Berube, Collin J. Popp, Margaret Curran, Mary Lou Pompeii, Lu Hu, Jose O. Aleman, Michael Bergman, Mary Ann Sevick

**Affiliations:** 1 Division of Precision Medicine, Department of Medicine, NYU Grossman School of Medicine, New York, New York, USA; 2 Department of Epidemiology, Public Health Nutrition Program, NYU School of Global Public Health, New York, New York, USA; 3 Department of Population Health, NYU Grossman School of Medicine, New York, New York, USA; 4 Holman Division of Endocrinology, Department of Medicine, NYU Grossman School of Medicine, New York, New York, USA

**Keywords:** type 2 diabetes

## Abstract

**Aims:**

To characterize changes in continuous glucose monitoring (CGM)-derived time in tight range (TIR) measures in individuals with prediabetes or non-insulin-treated type 2 diabetes undergoing dietary weight loss intervention and to quantify the association between weight loss and TIR improvement.

**Methods:**

Data from the Personal Diet Study, a 6-month behavioural weight loss intervention in adults with prediabetes or non-insulin-treated type 2 diabetes [HbA_1c_ ≤ 8.0% (64 mmol/mol), managed with diet alone or with metformin], was analysed. Participants wore a CGM for a maximum of 2 weeks at baseline and 6 months. Changes in overall, daytime (06:00 h–23:59 h) and overnight (00:00 h–05:59 h) time in 54–140 mg/dL or 3.0–7.8 mmol/L (TIR_54–140_), 70–140 mg/dL or 3.9–7.8 mmol/L (TIR_70–140_) and >140 mg/dL or >7.8 mmol/L (TAR_>140_) were analysed. The association between weight change and TIR change adjusted for demographic and clinical covariates was computed using linear regression.

**Results:**

Baseline and 6 months CGM data from 76 participants (63 ± 8 years, 62% female, 64% White, BMI 33 ± 5 kg/m^2^, HbA_1c_ 5.8 ± 0.6%) were analysed. Overall TIR_54–140_ increased (3.3% [0.3, 6.3]%; *p* = 0.03), with improvement in daytime (3.8% [0.9, 6.8]%; *p* = 0.01) but not overnight TIR_54–140_ (2.0% [−2.2, 6.1]%; *p* = 0.36). In adjusted analysis, every 5% points of weight loss was associated with a 3.2% points increase in overall TIR_54–140_ (*p* = 0.016), driven by a 3.5% points increase in daytime TIR_54–140_ (*p* = 0.006). Similar associations were found for TAR_>140_ but not TIR_70–140_. There were no associations between weight loss and change in any overnight TIR measure.

**Conclusion:**

Weight loss was associated with improved daytime TIR_54–140_ and TAR_>140_ in individuals with prediabetes and non-insulin-treated type 2 diabetes undergoing dietary intervention. The daytime time in tight range measures can complement traditional markers like HbA1c, offering a more comprehensive view of glycaemic variations during dietary weight loss programmes for individuals with prediabetes and type 2 diabetes not on insulin.

## 1 | INTRODUCTION

Continuous glucose monitoring (CGM) is beneficial for the management of type 1 diabetes and insulin-treated type 2 diabetes.^1–3^ The 2023 International Consensus Statement for CGM use recommended CGM-derived measures to complement traditional outcomes such as HbA_1c_ to evaluate the effectiveness of clinical trials for diabetes management.^4^ However, the utility of CGM-derived measures in prediabetes or non-insulin-treated type 2 diabetes management is unclear. Time in range (TIR) measures have emerged as a crucial CGM-derived measure linked with diabetes-related complications in type 1 diabetes and insulin-treated type 2 diabetes.^4–7^ Yet, few studies have examined TIR measures in individuals with prediabetes or non-insulin-treated type 2 diabetes undergoing dietary weight loss interventions. In this study, we examined TIR changes in adults with prediabetes or type 2 diabetes managed with diet alone or with metformin during a 6-month weight loss intervention.

Recent research suggests TIR measures like time in 54–140 mg/dL (3.0–7.8 mmol/L; TIR_54–140_), time in 70–140 mg/dL (3.9–7.8 mmol/L; TIR_70–140_), and time above 140 mg/dL (>7.8 mmol/L; TAR_>140_), also referred to as time in tight range measures,^8^ may capture dysglycemia in prediabetes and non-insulin-treated type 2 diabetes better than the conventional time in 70–180 mg/dL (3.9–10 mmol/L; TIR_70–180_) measure used in the management of type 1 diabetes and insulin-treated type 2 diabetes.^9,10^ A previous study reported that individuals with prediabetes and non-insulin-treated T2D spent on average 98% and 86% of time within the 70–180 mg/dL range (3.9–10 mmol/L), and 92% and 60% of time within 70–140 mg/dL (3.9–7.8 mmol/L), respectively.^9^ Another study in individuals with prediabetes found TIR_70–180_ and TIR_70–140_ values to be 99% and 90% respectively.^11^ Consequently, the TIR_70–180_ being close to 100% in this population is likely to miss small yet clinically significant changes (≥5%),^8^ whereas the TIR_70–140_ metric is more capable of detecting such variations. A review examining TIR data from 16 studies in people with prediabetes recommended TIR_54–140_ as an appropriate target range, as a key goal of this population is to achieve glucose levels as close to normoglycemia (54–140 mg/dL or 3.0–7.8 mmol/L) as possible.^12^ A study in Hispanic/Latino adults with prediabetes and non-insulin-treated type 2 diabetes found that splitting TIR_70–140_ and TAR_>140_ into daytime and overnight components offered new insights into glucose dynamics,^9^ with daytime TIR measures revealing a large degree of dysglycemia while overnight TIR measures were similar to normoglycemia. Therefore, examining changes in TIR_54–140_, TIR_70–140_, and TAR_>140_ in individuals with prediabetes and non-insulin-treated type 2 diabetes undergoing dietary intervention could provide valuable information about the intervention’s effectiveness.

The present analysis is based on the Personal Diet Study,^13^ a 6-month behavioural weight loss intervention in adults with prediabetes and non-insulin-treated type 2 diabetes who wore a blinded CGM at the start of and at the conclusion of the trial. The current analysis had two objectives: first, we profiled changes in TIR_54–140_, TIR_70–140_, and TAR_>140_ over the 6-month intervention. Second, we examined the association of weight loss and TIR improvement, adjusted for known demographic and clinical covariates.

## 2 | RESEARCH DESIGN AND METHODS

We performed a secondary analysis of CGM data from the Personal Diet Study,^13^ a technology-based behavioural weight loss intervention in individuals with prediabetes and non-insulin-treated type 2 diabetes. The Personal Diet Study was approved by the Institutional Review Board of NYU Grossman School of Medicine (IRB #17-00741). All participants provided written informed consent.

### 2.1 | Study design

The Personal Diet Study was a 2-phase, parallel-group randomized clinical trial consisting of 6 months of active intervention, details of which have been previously published.^13,14^ Participants were primarily recruited from NYU Langone Health and its affiliates in the period February 12, 2018 to October 28, 2021. They were equally randomized to a Standardized or Personalized diet arm. Participants in both arms received behavioural weight loss counselling, a caloric reduction target of 500 kcal/day, and recorded dietary intake using the Personalized Nutrition Project (PNP) smartphone app. The Standardized arm was counselled to follow a low-fat diet (<25% energy intake from dietary fat) and received standard feedback from the PNP app concerning calories and macronutrient distribution. The Personalized arm received the same PNP app feedback as the Standardized arm, in addition to personalized feedback on predicted postprandial glycaemic response generated from a gut microbiome-based machine learning algorithm.^15^ Participants were instructed to wear the Abbott FreeStyle Libre Pro (Abbott Park, IL, USA), a blinded CGM that measures interstitial glucose every 15 min, for up to 14 days at baseline and at 6 months. Weight data were also recorded at baseline and 6 months using validated weight scales (Stow-A-Weigh Scale-Tronix; Welch Allyn and Renpho, details published previously^13^).

### 2.2 | Recruitment

Participants were recruited from the NYU Langone Health system using MyChart patient messaging. Eligibility criteria included English-speaking adults aged 18 to 80 years, with overweight or obesity (defined as body mass index [BMI] ≥27 kg/m^2^ and ≤ 50 kg/m^2^) and prediabetes or type 2 diabetes with an HbA_1c_ between 5.7%–8.0% (39–64 mmol/mol) managed with lifestyle alone or lifestyle plus metformin. Detailed inclusion and exclusion criteria have been published previously.^13,14^

### 2.3 | Outcomes

The primary outcome for this analysis was the change in TIR_54–140_, while the change in TIR_70–140_ and TAR_>140_ were the secondary outcomes. We focused on the former as the primary outcome because previous studies show that asymptomatic hypoglycemic readings between 54 and 70 mg/dL (3.0–3.9 mmol/L) are common in individuals with prediabetes and non-insulin-treated type 2 diabetes.^12,16^ Therefore, TIR_54–140_ can better capture “in-range” glucose values than TIR_70–140_ in this population.^17^ We studied changes in these TIR measures using: (1) all glucose measurements (henceforward “Overall-TIR”), (2) daytime glucose measurements between 06:00 h and 23:59 h (henceforward “Daytime-TIR”), and (3) overnight glucose measurements between 00:00 h and 05:59 h (henceforward “Overnight-TIR”), following international CGM guidelines.^8^ We removed the first 12 h of CGM data as per Freestyle Libre guidelines due to the potential for low accuracy.^18^ TIR measures for each participant at baseline and 6 months were calculated as a percentage of glucose measurements in the target range with respect to the total number of measurements.^8^

### 2.4 | Statistical analysis

Change in TIR measures was computed as per CGM consensus guidelines^8^ as the baseline value of a given TIR measure subtracted from the 6-month value of that TIR measure, and reported as percentage points. For example, if a participant’s daytime TIR_54–140_ increased from 70% to 80% from baseline to 6 months, their change in TIR was recorded as 10% points. Statistical significance of the difference in TIR values between baseline and 6 months was computed using a *t*-test. Next, we performed linear regression analyses with change in a given TIR measure as the outcome and percent weight change as the exposure, adjusted for study arm (Standardized or Personalized diet), age, self-reported sex, race and ethnicity, baseline BMI, metformin use, and baseline TIR. The appropriate baseline TIR measure was used in a given regression model depending on the glucose range (TIR_54–140_, TIR_70–140_, or TAR_>140_) and timing of glucose measurements used (Overall, Daytime or Overnight). For instance, the regression model with change in Daytime TIR_54–140_ as the outcome was adjusted for the baseline Daytime TIR_54–140_ values for each participant. Statistical analyses were performed using the “Statsmodels” and “Scipy” packages in Python,^19^ and “stats” package in R.^20^ Statistical significance was assessed using a threshold of alpha = 0.05.

## 3 | RESULTS

### 3.1 | Participant characteristics

We analyzed data from a subset of 76 participants in the Personal Diet Study who had CGM data at baseline and at 6 months. Participants were aged 63 ± 8 years, 62% female, 64% White, 20% African American, and had a baseline BMI of 33 ± 5 kg/m^2^ and a baseline HbA_1c_ of 5.8 ± 0.5%. On average, participants had 7.1 ± 2.0 days and 7.2 ± 1.9 days of analyzable CGM data at baseline and 6 months, respectively. Detailed participant characteristics are reported in Table 1.

### 3.2 | Descriptive analysis of TIR measures

We first performed a descriptive analysis for the change in each TIR measure of interest from baseline to 6 months (Table 2). There was a 3.3-point increase in Overall TIR_54–140_ (*p* = 0.035), driven by a 3.8-point increase in Daytime TIR_54–140_ (*p* = 0.013). Similarly, there was a 3.5-point decrease in Overall TAR_>140_, driven by a 3.8-point decrease in Daytime TAR_>140_ (*p* = 0.008). No statistically meaningful change in TIR_70–140_ was observed (all *p* > 0.1). Notably, none of the overnight TIR measures had a significant change from baseline to 6 months (all *p* > 0.2).

The CGM consensus guidelines identified a change of 5% points of TIR as clinically meaningful.^8^ Using this threshold, 19 (25%) participants had a clinically meaningful improvement in Daytime TIR_54–140_ and 17 (22.4%) in Overall TIR_54–140_. On the other hand, 19 (25%) participants had a clinically meaningful reduction in Daytime TAR_>140_ and 15 (19.7%) in Overall TAR_>140_. Detailed information on clinically meaningful changes in all TIR measures is also included in Table S1.

### 3.3 | Linear regression to quantify the association of weight loss and TIR improvement

We then quantified the association of percent weight change and change in TIR measures from baseline to 6 months using linear regression. We performed the regression analyses only with Overall and Daytime TIR_54–140_ and TAR_>140_, as these four TIR measures were the only ones that changed significantly, as shown in Table 2.

**FIGURE 1:**
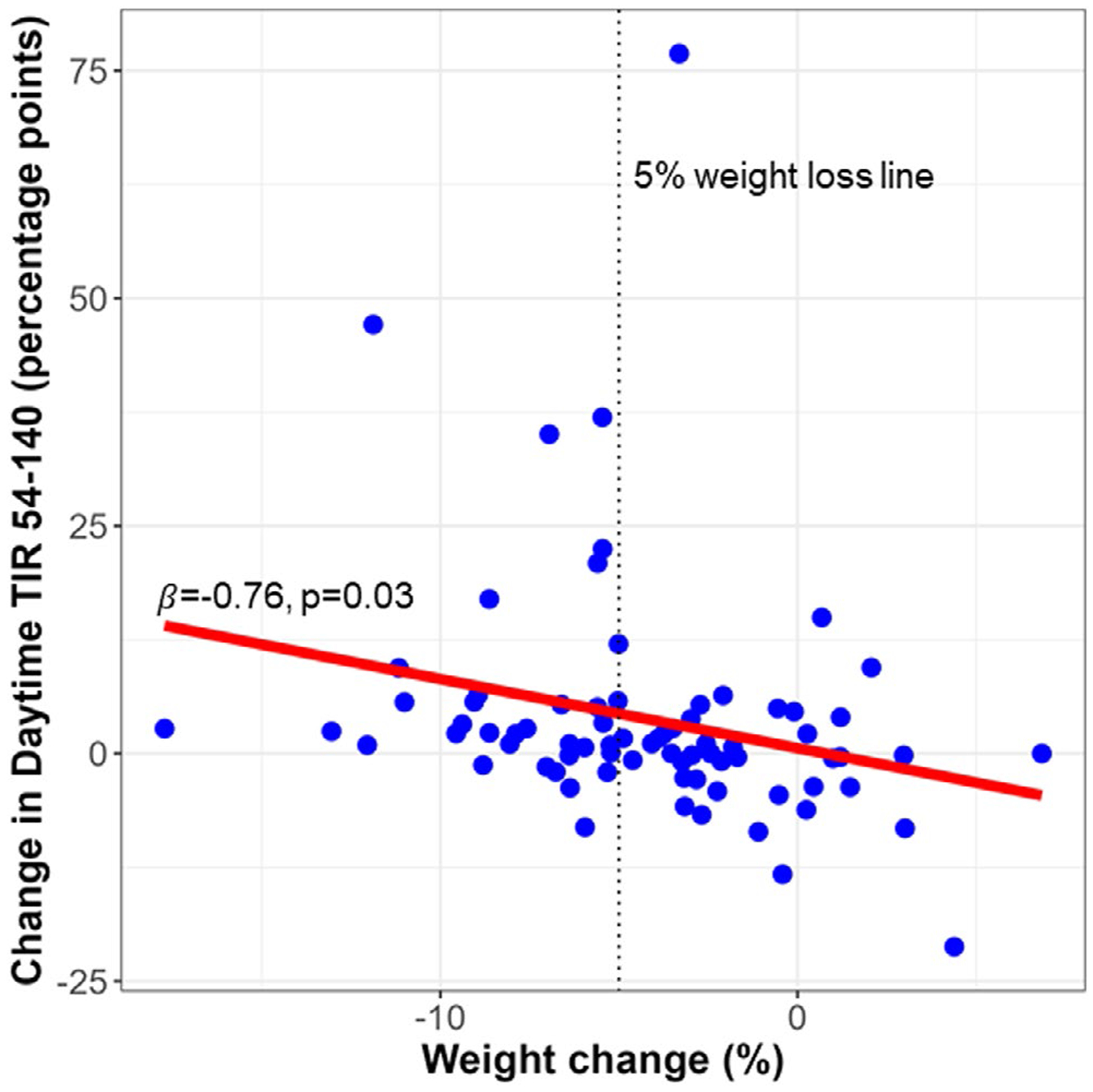
Scatter plot of change in weight versus change in Daytime TIR_54–140_ from baseline to 6 months (*n* = 76). The vertical dotted line represents a clinically meaningful 5% weight loss. The red line represents the best-fit line, with the regression coefficient (*β*) and *p*-value noted. Positive value for weight change represents weight gain and negative value represents weight loss over the 6-month intervention. Positive value for change in Daytime TIR_54–140_ represents improvement while negative value represents decline in time spent in the 54–140 mg/dL range during daytime over the course of the intervention. TIR_54–140_: Time in 54–140 mg/ dL (3.0–7.8 mmol/L) range.

*TIR_54–140_* : In univariate analysis, weight loss was associated with increased Daytime TIR_54–140_ (*p* = 0.030) and Overall TIR_54–140_ (*p* = 0.041) (Figure 1, Table S2). After adjusting for covariates including the intervention arm, weight loss was independently associated with increased Daytime TIR_54–140_ (*β* = −0.70, 95% CI: [−1.19, −0.21]; *p* = 0.006) (Table 3). Weight loss was also independently associated with increased Overall TIR_54–140_ (*β* = −0.64, 95% CI: [−1.14, −0.13]; *p* = 0.016) (Table S3). These findings suggest that a clinically meaningful 5% weight loss was associated with an average 3.5 and 3.1% point, or equivalently 50 and 45 min increase in Daytime and Overall TIR_54–140_, respectively. Amongst covariates, baseline TIR values were significantly associated with change in TIR values in all analyses. Individuals with lower baseline TIR_54–140_ or higher TAR > 140 had the greatest improvement in TIR measures over the 6-month intervention.

*TAR_>140_* : In univariate analysis, weight loss had a weak association with change in daytime TAR_>140_ (*p* = 0.089) and overall TAR_>140_ (*p* = 0.088) (Figure S1, Table S2). In adjusted multivariate analysis, weight loss was independently associated with decreased Daytime TAR_>140_ (*β* = 0.50, 95% CI: [0.04, 0.96]; *p* = 0.037) and weakly associated with increase in Overall TAR_>140_ (*β* = 0.47, 95% CI: [−0.01, 0.94]; *p* = 0.06). Again, lower baseline TAR_>140_ had a significant association with increased Daytime as well as Overall TAR_>140_ (Tables S4 and S5).

## 4 | CONCLUSIONS

The value of CGM-derived measures, such as TIR, is unclear for individuals with prediabetes and non-insulin-treated type 2 diabetes managed with diet alone or with metformin. In this study, we investigated changes in the CGM-based TIR_54–140_, TIR_70–140_, and TAR_>140_ measures over the course of a 6-month dietary intervention in this population. We observed significant improvement in Overall TIR_54–140_ and TAR_>140_. This improvement was driven predominantly by improvement in Daytime TIR_54–140_ and TAR_>140_, with no meaningful improvement in Overnight TIR_54–140_ and TAR_>140_.

Studies have shown that weight loss improves TIR in people with type 2 diabetes undergoing pharmacological interventions, such as SGLT2 or GLP-1 medications.^21,22^ However, there is minimal existing data on TIR changes in individuals with prediabetes or with non-insulin-treated type 2 diabetes undergoing dietary weight loss intervention. A pilot study in 15 individuals with prediabetes and obesity receiving a low-carbohydrate diet while simultaneously wearing a CGM showed small decreases in weight (1%) and in overall time above 140 mg/dL or 7.8 mmol/L (2.6%).^23^ Another study in 20 adults with overweight or obesity and HbA_1c_ 6%–7% receiving a low-carbohydrate diet showed a slight non-significant decrease in overall time above 180 mg/dL.^24^ Two digital health interventions for weight loss in people with type 2 diabetes only (January AI study) and prediabetes and type 2 diabetes (Fitterfly Diabetes CGM program) respectively showed significant weight loss and improvement in TIR_70–180_, although the correlation between the two was not reported.^25,26^ In this study, we observed small but significant improvements in Overall TIR_54–140_ and TAR_>140_. However, we did not find studies examining similar effects on Daytime TIR_54–140_ and TAR_>140_, which our study contributes to the literature.

We also examined the association of change in weight and our chosen TIR measures from baseline to the end of the intervention at 6 months, to quantify the relationship between weight loss and glycemic improvement. In multivariate analysis, we observed that weight loss was independently associated with improvement in Overall and Daytime TIR_54–140_. In particular, our analysis suggests that a clinically significant 5% point weight loss^27^ was on average associated with a 3.5-point improvement, or a 50-min daily increase in time spent in the 54–140 mg/dL range during daytime. Similar but weaker associations were observed between weight loss and improvement in Overall and Daytime TAR_>140_. The findings for TAR_>140_ being similar but not identical may be attributed to sensor noise especially at lower glucose readings, which is known to affect TIR measures.^28,29^ Daytime TIR is significantly influenced by postprandial glucose response,^9^ which may indicate an association between reduction in postprandial glucose excursions and weight loss in this population. Furthermore, prior studies have shown that large postprandial glucose excursions may contribute more to β-cell damage than steady-state hyperglycemia.^30,31^ The 1-h postprandial glucose, measured by CGM, has been shown to be associated with overall dysglycemia and glycemic variability.^32,33^ In addition, weight loss has been associated with improved insulin sensitivity and β-cell function.^34^ Therefore, improvement in Daytime TIR may be a surrogate marker of improvement in β-cell function and peripheral insulin sensitivity^35^ after dietary interven- tion—although this needs evaluation in future studies. While the lack of similar improvement in Overnight TIR could be explained by minimal improvement in hepatic insulin sensitivity,^35^ this could also be due to our sample having high Overnight TIR to begin with, leaving little room for improvement. Larger studies incorporating CGM assessments with concurrent fasting and postprandial insulin measurements in this population could illuminate how specific types of insulin sensitivity (e.g. peripheral vs. hepatic) relate to daytime/overnight TIR measures and weight loss. Overall, our work provides much-needed data on the extent of glycemic improvement corresponding to a clinically significant 5% weight loss, which can inform glycemic targets for future diet-focused weight loss trials in individuals with prediabetes and non-insulin-treated type 2 diabetes.

The current study had multiple strengths and limitations. Strengths included the use of free-living CGM data, which allowed a detailed interrogation of glycemic changes from baseline to the end of the study at 6 months using multiple TIR measures. Another strength was that the study used a blinded CGM, ensuring that improvement in TIR was not biased by visualization of CGM data. Limitations included a relatively small sample size due to the COVID-19 pandemic and a limited period of glucose monitoring (average 7 days) compared to CGM guidelines (14 days).^8^ The generalizability of our findings needs to be examined in a larger and more diverse cohort with longer CGM wear times. We were not able to account for physical activity during the study period, which may have contributed to changes in Daytime TIR in addition to dietary intervention.^36,37^ We also did not record any sleep information, which would have enabled the Daytime and Overnight TIR measures to reflect glycemic patterns more precisely during awake and sleep periods. Further, around 74% of participants had a baseline overnight TAR_>140_ less than 5%, so this measure may not be able to capture nighttime glycemic improvements over the course of the 6-month intervention in this cohort. Finally, we did not have data on actual dietary choices made by participants during the 6-month intervention, which could provide a better understanding of causal factors involved in the improvement in Daytime TIR measures viz. a viz. weight loss. A closer interrogation of why the different dietary arms did not show differences in improvement in TIR, adjusted for weight change, is also warranted in future studies, including factors such as adherence to dietary recommendations and the effect of under-reporting of dietary intake. Future studies with detailed data on dietary choices and physical activity/sleep patterns may provide a more comprehensive understanding of the diet, weight loss, and glucose nexus.

In summary, this study characterized changes in CGM-based TIR measures to assess glycemic improvements in individuals with prediabetes and non-insulin-treated type 2 diabetes undergoing a 6-month dietary intervention. We showed that the time spent in 54–140 mg/dL and time above 140 mg/dL during the daytime (06:00 h–23:59 h) may better capture reductions in postprandial glucose excursions and better explain weight loss over the course of a dietary intervention than TIR measures computed using all or overnight glucose readings. Our findings highlight Daytime TIR_54–140_ and TAR_>140_ as potential CGM-based endpoints to complement traditional glycemic endpoints such as HbA_1c_ for dietary interventions in individuals with prediabetes and non-insulin-treated type 2 diabetes.

## Supplementary Material

Barua_DME_Supp

Additional supporting information can be found online in the Supporting Information section at the end of this article.

## Figures and Tables

**TABLE 1:** Participant characteristics at baseline (*n* = 76).

Variable	Value
Number of participants	76
Age (in years)	62 ± 9
Self-reported sex	
• Female	50 (66%)
• Male	26 (34%)
Self-reported race	
• White	49 (64%)
• Black or African—American	13 (17%)
• Other	14 (19%)
Self-reported ethnicity	
• Not Hispanic or Latino	65 (86%)
• Hispanic or Latino	11 (14%)
BMI (kg/m^2^)	33.2 ± 5.0
Metformin use	
• Yes	15 (20%)
• No	61 (80%)
HbA_1c_ (%)	5.8 ± 0.6
Self-reported diagnosis of type 2 diabetes	15 (20%)
Days of CGM data	
• Baseline	7.1 ± 2.0
• 6 months	7.2 ± 1.9

*Note*: Values reported as mean ± SD or as a percentage for all participants.

**TABLE 2:** Descriptive analysis for change in each TIR measure of interest (*n* = 76).

TIR measure	Baseline (%)	6-months (%)	Change (%)	*p*
TIR_54–140_				
• Overall	89.6 [86.1, 93.1]	92.9 [90.5, 95.3]	**3.3 [0.3, 6.3]**	**0.034**
• Daytime	88.7 [85.0, 92.3]	92.5 [90.0, 95.0]	**3.8 [0.9, 6.8]**	**0.013**
• Overnight	92.1 [88.4, 95.7]	94.0 [91.1, 97.0]	2.0 [−2.2, 6.1]	0.361
TIR_70–140_				
• Overall	85.7 [82.0, 89.5]	87.8 [84.8, 90.8]	2.0 [−1.5, 5.6]	0.257
• Daytime	85.2 [81.4, 89.1]	87.9 [85.0, 90.8]	2.7 [−0.7, 6.0]	0.121
• Overnight	87.0 [82.9, 91.1]	87.3 [83.4, 91.1]	0.3 [−4.7, 5.2]	0.921
TAR_>140_				
• Overall	9.1 [5.6, 12.7]	5.7 [3.3, 8.1]	**−3.5 [−6.3, −0.6]**	**0.020**
• Daytime	10.3 [6.7, 13.9]	6.5 [4.0, 8.9]	**−3.8 [−6.6, −1.1]**	**0.008**
• Overnight	6.0 [2.5, 9.5]	3.6 [0.9, 6.3]	−2.4 [−6.2, 1.4]	0.217

*Note*: Change in TIR measure defined as value at baseline subtracted from value at 6 months. Values reported as mean [95% CI]. TIR_54–140_: Time in 54–140 mg/dL (3.0–7.8 mmol/L). TIR_70–140_: Time in 70–140 mg/dL (3.9–7.8 mmol/L). TAR_>140_: Time above 140 mg/dL (7.8 mmol/L). Statsitical significance was assessed for *p* < 0.05.

Abbreviations: CI, confidence interval; TIR, time in range.

**TABLE 3:** Multivariate linear regression to quantify the association between weight change and change in Daytime TIR_54–140_, adjusted for known demographic and clinical covariates (*n* = 76).

Variable	Coefficient estimate (95% CI)	*p*-value
Intercept	4.85 (0.64, 9.07)	0.027
Arm (ref: Standardized arm)		
• Personalized arm	0.47 (−3.56, 4.49)	0.820
Age	0.09 (−0.14, 0.32)	0.425
Self-reported sex (ref: Female)		
• Male	−2.81 (−7.80, 2.18)	0.274
Self-reported race (ref: White)		
• Black or African-American	1.13 (−4.64, 6.90)	0.703
• Other	1.20 (−4.18, 6.59)	0.663
Self-reported ethnicity (ref: Not Hispanic or Latino)		
• Hispanic or Latino	0.38 (−5.53, 6.28)	0.901
BMI	0.08 (−0.34, 0.50)	0.716
Metformin use (ref: No)		
• Yes	−3.98 (−9.41, 1.45)	0.156
**% Weight change**	**−0.70 (−1.19, −0.21)**	**0.006**
**Baseline Daytime TIR_54–140_**	**−0.67 (−0.82, −0.53)**	**<0.0001**
TIR_54–140_: Time in 54–140 mg/dL (3.0–7.8 mmol/L) glucose range		

*Note*: TIR_54–140_: Time in 54–140 mg/dL (3.0–7.8 mmol/L). Statistical significance was assessed for *p*<0.05.

Abbreviations: BMI, body mass index; CI, confidence interval.

## Data Availability

The dataset and code required to reproduce the findings in this study may be made available by the corresponding author upon reasonable request to mary.sevick@nyulangone.org and souptik.barua@nyulangone.org, respectively.

## What’s new?

- *What is already known on this topic?* The value of continuous glucose monitoring (CGM)- based time in range (TIR) measures for the management of type 1 and insulin-treated type 2 diabetes has been established through several studies. However, limited evidence exists regarding the value of TIR measures in the management of prediabetes and non-insulin-treated type 2 diabetes.
- *What this study adds?* We perform a comprehensive characterization of TIR measures in individuals with prediabetes and non-insulin- treated type 2 diabetes undergoing a 6-month dietary weight loss intervention. We observed a significant association between weight loss and improvement in time in 54–140 mg/dL (3.0–7.8 mmol/L) and time above 140 mg/dL (7.8 mmol/L) during daytime (06:00–23:59 h) but not during overnight (00:00–05:59 h).
- *How this study might affect research, practice or policy?* Daytime TIR measures can complement traditional endpoints such as HbA_1c_ to provide a more complete picture of glycaemic changes during weight loss interventions in individuals with prediabetes and non-insulin-treated type 2 diabetes.
