# Supplementary material for: Weight loss is associated with improved daytime time in range in adults with prediabetes and non-insulin-treated type 2 diabetes undergoing dietary intervention
Source: Diabet Med. Author manuscript; Available in PMC 2026 May 3. (PMC13135705; doi:10.1111/dme.70052)
Supplement: Barua_DME_Supp [file NIHMS2166437-supplement-Barua_DME_Supp.docx]

**SUPPLEMENTARY DATA**

**Supplementary Table 1: Descriptive analysis for change in each TIR measure of interest for participant subgroups stratified by a clinically meaningful 5-point change in TIR measures. *Improved*: Participants TIR had a clinically meaningful improvement of 5 percentage points or more; *Declined*: Participants TIR had a clinically meaningful decline of 5 percentage points or more; *No Change*: Participants whose TIR had an absolute change of less than 5 percentage points (n=76). TIR_54-140_: Time in 54-140 mg/dL (3.0-7.8 mmol/L) glucose range, TAR_>140_: Time above 140 mg/dL (7.8 mmol/L) glucose range, TIR_70-140_: Time in 70-140 mg/dL (3.9-7.8 mmol/L) glucose range.**

| **TIR change measure** | **Number of participants (%)** |
| --- | --- |
| TIR_54-140_   - Overall - Daytime - Overnight | 17 (22.4%) Improved  49 (64.5%) No Change  10 (13.2%) Declined  19 (25.0%) Improved  49 (64.5%) No Change  8 (10.5%) Declined  15 (19.7%) Improved  52 (68.4%) No Change  9 (11.8%) Declined |
| TAR_>140_   - Overall - Daytime - Overnight | 15 (19.7%) Improved  56 (73.7%) No Change  5 (6.6%) Declined  19 (25.0%) Improved  52 (68.4%) No Change  5 (6.6%) Declined  12 (15.8%) Improved  61 (80.3%) No Change  3 (3.9%) Declined |
| TIR_70-140_   - Overall - Daytime - Overnight | 18 (23.7%) Improved  43 (56.6%) No Change  15 (19.7%) Declined  22 (28.9%) Improved  37 (48.7%) No Change  17 (22.4%) Declined  24 (31.6%) Improved  29 (38.2%) No Change  23 (30.3%) Declined |

**Supplementary Table 2: Univariate linear regression analysis with %weight change as exposure and change in a given TIR measure as outcome (n=76). Each row reports regression coefficient estimates and p-values from the linear regression model for the respective TIR outcome. CI: Confidence interval, BMI: Body mass index, TIR_54-140_: Time in 54-140 mg/dL (3.0-7.8 mmol/L) glucose range, TAR_>140_: Time above 140 mg/dL (7.8 mmol/L) glucose range.**

| **TIR outcome** | **Coefficient estimate (95% CI) for %weight change** | **p-value** |
| --- | --- | --- |
| TIR_54-140_   - Overall - Daytime - Overnight | -0.73 (-1.42,-0.04)  -0.76 (-1.43,-0.09)  -0.67 (-1.62,0.28) | 0.041  0.030  0.173 |
| TAR_>140_   - Overall - Daytime - Overnight | 0.58 (-0.08,1.23)  0.55 (-0.07,1.18)  0.64 (-0.24,1.53) | 0.089  0.089  0.157 |

**Supplementary Table 3: Multivariate linear regression to quantify association between %weight change and change in Overall** **TIR_54-140_, adjusted for known demographic and clinical covariates (n=76). CI: Confidence interval, BMI: Body mass index, TIR_54-140_: Time in 54-140 mg/dL (3.0-7.8 mmol/L) range.**

| **Variable** | **Coefficient estimate (95% CI)** | **p-value** |
| --- | --- | --- |
| Intercept | 4.90 (0.59,9.21) | 0.029 |
| Arm (ref: Standardized arm)   - Personalized arm | -0.25 (-4.39,3.89) | 0.906 |
| Age | 0.14 (-0.10,0.37) | 0.257 |
| Self-reported sex (ref: Female)   - Male | -3.66 (-8.75,1.44) | 0.164 |
| Self-reported race (ref: White)   - Black or African-American - Other | 0.75 (-5.17,6.67)  2.02 (-3.51,7.55) | 0.805  0.477 |
| Self-reported ethnicity (ref: Not Hispanic or Latino)   - Hispanic or Latino | -0.07 (-6.13,5.99) | 0.982 |
| Baseline BMI | 0.06 (-0.37,0.49) | 0.793 |
| Metformin use (ref: No)   - Yes | -3.52 (-9.12,2.08) | 0.222 |
| **% Weight change** | **-0.62 (-1.12,-0.11)** | **0.019** |
| **Baseline Overall TIR_54-140_** | **-0.72 (-0.87,-0.56)** | **<0.0001** |

**Supplementary Table 4: Multivariate linear regression to quantify association between weight change and change in Daytime** **TAR_>140_, adjusted for known demographic and clinical covariates (n=76). CI: Confidence interval, BMI: Body mass index, TAR_>140_: Time above 140 mg/dL (7.8 mmol/L) range.**

| **Variable** | **Coefficient estimate (95% CI)** | **p-value** |
| --- | --- | --- |
| Intercept | -3.76 (-16.49,8.97) | 0.565 |
| Arm (ref: Standardized arm)   - Personalized arm | 0.39 (-3.39,4.17) | 0.841 |
| Age | -0.17 (-0.39,0.04) | 0.120 |
| Self-reported sex (ref: Female)   - Male | 2.66 (-1.92,7.23) | 0.260 |
| Self-reported race (ref: White)   - Black or African-American - Other | -0.53 (-5.93,4.86)  -2.92 (-7.95,2.11) | 0.847  0.260 |
| Self-reported ethnicity (ref: Not Hispanic or Latino)   - Hispanic or Latino | -0.29 (-5.82,5.25) | 0.919 |
| Baseline BMI | -0.04 (-0.43,0.36) | 0.857 |
| Metformin use (ref: No)   - Yes | 3.54 (-1.59,8.68) | 0.181 |
| **% Weight change** | **0.49 (0.03,0.94)** | **0.041** |
| **Baseline Daytime TAR_>140_** | **-0.62 (-0.76,-0.49)** | **<0.0001** |

**Supplementary Table 5: Multivariate linear regression to quantify association between weight change and change in Overall** **TAR_>140_, adjusted for known demographic and clinical covariates. (n=76). CI: Confidence interval, BMI: Body mass index, TAR_>140_: Time above 140 mg/dL (7.8 mmol/L) range.**

| **Variable** | **Coefficient estimate (95% CI)** | **p-value** |
| --- | --- | --- |
| Intercept | -4.67 (-8.72,0.62) | 0.027 |
| Arm (ref: Standardized arm)   - Personalized arm | 1.05 (-2.88,4.98) | 0.602 |
| Age | -0.20 (-0.43,0.02) | 0.078 |
| Self-reported sex (ref: Female)   - Male | 3.03 (-1.70,7.77) | 0.214 |
| Self-reported race (ref: White)   - Black or African-American - Other | -0.97 (-6.57,4.62)  -3.54 (-8.76,1.67) | 0.734  0.188 |
| Self-reported ethnicity (ref: Not Hispanic or Latino)   - Hispanic or Latino | -0.34 (-6.07,5.39) | 0.908 |
| Baseline BMI | 0.03 (-0.39,0.44) | 0.905 |
| Metformin use (ref: No)   - Yes | 2.52 (-2.81,7.85) | 0.357 |
| **% Weight change** | **0.47 (0.00,0.95)** | **0.054** |
| **Baseline Overall TAR_>140_** | **-0.66 (-0.81,-0.51)** | **<0.0001** |

**Supplementary Figure 1: Scatter plot of change in weight vs. change in Daytime TAR_>140_ from baseline to 6 months (n=76). The vertical dotted line represents a clinically meaningful 5% weight loss. The red line represents the best-fit line, with the regression coefficient (β) and p-value noted. Positive value for weight change represents weight gain and negative value represents weight loss over the 6-month intervention. Positive value for change in Daytime TAR_>140_ represents an increase in time above 140 mg/dL while negative value represents decrease in time above 140 mg/dL over the course of the intervention. TAR_>140_: Time above 140 mg/dL (7.8 mmol/L) range.**

**
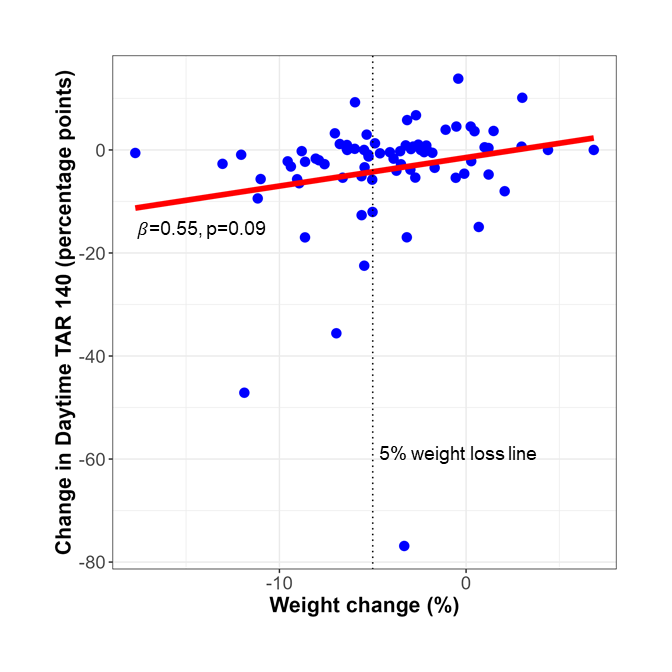
**
